# Rate of SARS-CoV-2 Reinfection During an Omicron Wave in Iceland

**DOI:** 10.1001/jamanetworkopen.2022.25320

**Published:** 2022-08-03

**Authors:** Elias Eythorsson, Hrafnhildur Linnet Runolfsdottir, Ragnar Freyr Ingvarsson, Martin I. Sigurdsson, Runolfur Palsson

**Affiliations:** 1 Landspitali–The National University Hospital of Iceland, Reykjavik, Iceland; 2 Faculty of Medicine, School of Health Sciences, University of Iceland, Reykjavik, Iceland

## Abstract

This cohort study estimates the proportion of persons who became reinfected with SARS-CoV-2 during the Omicron wave in Iceland.

## Introduction

COVID-19 caused by SARS-CoV-2 was declared a global pandemic on March 11, 2020. Omicron, the currently dominant variant, is characterized by increased immune evasion, making reinfections more common.^1,2^ The relative protection of prior infection against reinfection with Omicron is 56% compared with 92% for the Delta variant.^3^ However, the population-level risk of reinfection with Omicron has not been described. The aim of this study was to estimate the proportion of persons who become reinfected with SARS-CoV-2 during the Omicron wave in Iceland.

## Methods

The study was approved by the National Bioethics Committee of Iceland, which waived the need for informed consent. The study followed the Strengthening the Reporting of Observational Studies in Epidemiology (STROBE) reporting guideline. This population-based cohort study monitored all persons previously infected with SARS-CoV-2 for reinfection during the Omicron wave in Iceland, which was defined from December 1, 2021 (first diagnosed case of Omicron in Iceland) to the end of the study period on February 13, 2022. Data on all SARS-CoV-2 polymerase chain reaction (PCR) test results performed in the country were obtained from Landspitali–The National University Hospital of Iceland, and data on vaccine status were obtained from the Icelandic Directorate of Health. Reinfection was defined as a positive PCR test for SARS-CoV-2 60 or more days from a previous positive test. The proportion reinfected by age group, vaccine status, and the number of elapsed days from the initial positive PCR test was estimated using logistic regression. Statistical analyses were performed in R statistical software version 4.1.2 (R Project for Statistical Computing) using the *rms* package.^4^ Detailed definitions and all statistical codes are provided in eAppendix 1 and eAppendix 2 in the Supplement.

## Results

In total, 11 536 PCR-positive persons were included. The mean (SD) age was 34 (19) years (median, 31 years; range, 0-102 years), 5888 (51%) were male, 2942 (25.5%) had received at least 1 dose of vaccine, and the mean (SD) time from initial infection was 287 (191) days (median, 227 days; range, 60-642 days). Reinfection was observed in 1327 persons (11.5%) during the Omicron period. Of those who had received 1 dose or less of vaccine, 11.7% (1007 of 8598 individuals) were reinfected, compared with 10.9% (320 of 2938 individuals) who had received 2 or more doses. The reinfection rate was highest (475 of 3136 individuals [15.1%]) among those aged 18 to 29 years. Fewer reinfections occurred among older individuals (Table).

**Table.** Number and Proportion of Persons Reinfected With SARS-CoV-2 During the Omicron Wave of Infections in Iceland, December 1, 2021, to February 13, 2022

Variable	Reinfected individuals, No./total No. (%)	OR (95% CI)	
		Unadjusted	Adjusted^a^
Age group, y			
≤17	229/2113 (10.8)	0.68 (0.58-0.81)	0.81 (0.66-0.98)
18-29	475/3136 (15.1)	1 [Reference]	1 [Reference]
30-49	477/3724 (12.8)	0.82 (0.72-0.94)	0.79 (0.66-0.95)
50-74	136/2316 (5.9)	0.35 (0.29-0.43)	0.32 (0.24-0.44)
≥75	10/247 (4.1)	0.24 (0.15-0.45)	0.22 (0.08-0.61)
Sex			
Male	685/5888 (11.6)	1 [Reference]	NA
Female	642/5648 (11.3)	0.97 (0.87-1.09)	NA
Vaccine status			
≤1 Dose	1007/8598 (11.7)	1 [Reference]	1 [Reference]
≥2 Doses	320/2938 (10.9)	0.92 (0.81-1.05)	1.42 (1.13-1.78
Elapsed time from initial infection, mo			
≤3	338/3671 (9.2)	0.74 (0.65-0.85)	0.88 (0.65-1.20)
4-17	729/6082 (12.0)	1 [Reference]	1 [Reference]
≥18	260/1783 (14.6)	1.25 (1.08-1.46)	1.41 (1.05-1.90)

Abbreviations: NA, not applicable; OR, odds ratio.

^a^ The adjusted ORs are obtained from the logistic regression model and are adjusted to a reference individual aged 18 to 29 years who has received 1 dose or less of vaccine and whose previous infection occurred 227 days before December 1, 2021.

The probability of reinfection increased with time from the initial infection (odds ratio of 18 months vs 3 months, 1.56; 95% CI, 1.18-2.08) (Figure) and was higher among persons who had received 2 or more doses compared with 1 dose or less of vaccine (odds ratio, 1.42; 95% CI, 1.13-1.78). Defining reinfection after 30 or more days or 90 or more days did not qualitatively change the results.

**Figure.**
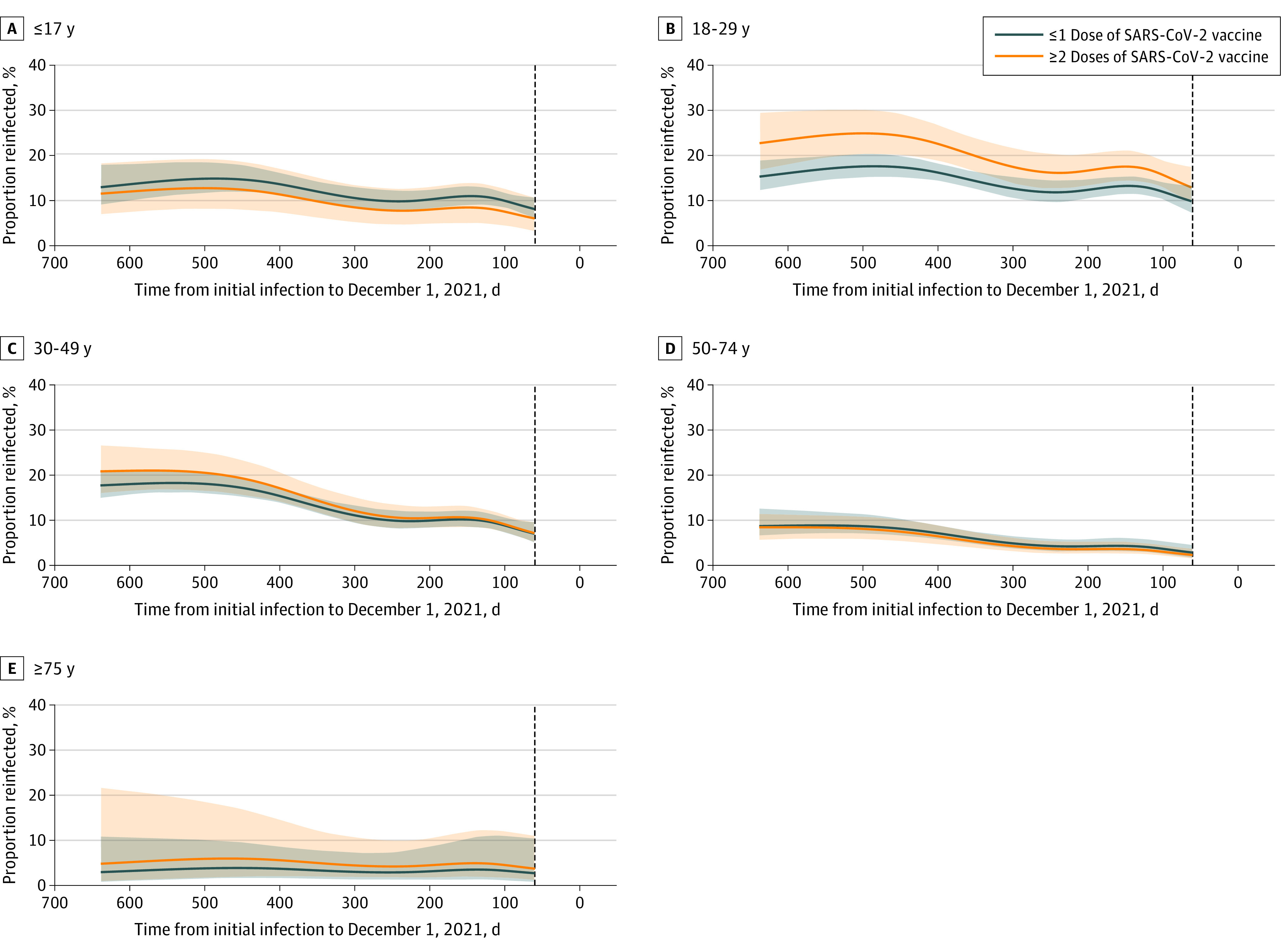
Proportion of Persons Reinfected With SARS-CoV-2 During the Omicron Wave of Infections in Iceland by Age Group, Vaccine Status, and Time From Initial Infection Shaded areas denote 95% CIs. A vertical dashed black line is drawn at 60 days. Persons whose initial infection occurred after that time were not at risk for reinfection on December 1, 2021, and were not included.

## Discussion

In this population-based cohort study, a substantial proportion of persons experienced SARS-CoV-2 reinfection during the first 74 days of the Omicron wave in Iceland, with rates as high 15.1% among those aged 18 to 29 years. Longer time from initial infection was associated with a higher probability of reinfection, although the difference was smaller than expected. Surprisingly, 2 or more doses of vaccine were associated with a slightly higher probability of reinfection compared with 1 dose or less. This finding should be interpreted with caution because of limitations of our study, which include the inability to adjust for the complex relationships among prior infection, vaccine eligibility, and underlying conditions. Importantly, by December 1, 2021, all persons aged 12 years and older were eligible for 2 or more vaccine doses free of charge, and 71.1% of the Icelandic population had been vaccinated,^5^ compared with only 25.5% of our cohort of previously infected persons. Our results suggest that reinfection is more common than previously thought. Now the key question is whether infection with the Omicron variant will produce better protection against Omicron reinfection, compared with other variants.

## Notes

Supplement. **eAppendix 1.** Supplemental Methods **eAppendix 2.** Statistical Codes
