# Supplementary material for: Rate of SARS-CoV-2 Reinfection During an Omicron Wave in Iceland
Source: JAMA Netw Open. 2022 Aug 3;5(8):e2225320. doi: 10.1001/jamanetworkopen.2022.25320 (PMC9350711; doi:10.1001/jamanetworkopen.2022.25320)
Supplement: Supplement. — eAppendix 1. Supplemental Methods eAppendix 2. Statistical Codes [file jamanetwopen-e2225320-s001.pdf]

Supplemental Online Content

Eythorsson E, Runolfsdottir HL, Ingvarsson RF, Sigurdsson MI, Palsson R. Rate of SARS-CoV-2 reinfection during an Omicron wave in Iceland. *JAMA Netw Open*. 2022;5(8):e2225320. doi:10.1001/jamanetworkopen.2022.25320

eAppendix 1. Supplemental Methods

eAppendix 2. Statistical Code

This supplemental material has been provided by the authors to give readers additional information about their work.

© 2022 Eythorsson E et al. *JAMA Network Open.*

eAppendix 1. Supplemental Methods

Data sources and definitions

The study population included all individuals who tested positive for SARS-CoV-2 by PCR in Iceland between February 28, 2020 and December 1, 2021. Three national testing programs were implemented during the study period; targeted testing based on clinical suspicion (from February 1, 2020), open invitation population screening (from March 13, 2020) and mandatory screening at the border (from June 15, 2020). All PCR-positive persons were prospectively followed at the Covid-19 Outpatient Clinic of Landspitali-The National University Hospital of Iceland, from which the data were obtained. For the main analysis, only those who had tested positive 60 days or longer before December 1, 2021 and had not died or been reinfected prior to that time were included. Sensitivity analyses were performed using definitions of 30 days or longer and 90 days or longer from the prior infection.

Vaccination against SARS-CoV-2 in Iceland began on December 28, 2021. Initially, only nursing home residents and front-line healthcare workers were vaccinated. By June 24, 2021, Covid-19 vaccines were available free of charge to all persons aged 16 years and older, and by June 28, 2021 vaccines were additionally offered to children 12-15 years of age. All persons 16 years of age and older who had received their second vaccine dose more than 6 months before were scheduled for a booster dose between November 15 and December 8, 2021. All administered doses of SARS-CoV-2 vaccines in Iceland until December 1, 2021 were obtained from the Icelandic Directorate of Health. We defined a person's vaccination status based on the number of vaccine doses he or she had received 14 days prior to December 1, 2021. Some combinations of age group and vaccine doses were extremely rare. Specifically, almost no persons aged 75 years and older had not received a single vaccine dose and few persons aged 17 years and younger and 18-29 years had received three or more doses. For this reason, we grouped together zero and one vaccine doses and two or more doses

Statistical modeling

The proportion of the study population that was reinfected between December 1, 2021 and February 13, 2022 was estimated using logistic regression by age group, vaccine status and a non-linear function of the number of elapsed days from the initial positive PCR test. Time from the initial positive PCR test was modeled using a five-knot restricted cubic spline (with knots placed at the 0.05, 0.275, 0.5, 0.725 and 0.95 percentiles), and linear interactions were allowed between age group, vaccine status and elapsed time from prior infection. The same logistic regression model was used to obtain adjusted odds ratios of reinfection, as compared to a person aged 18-29 years who had received ≤1 dose of vaccine and had experienced the first SARS-CoV-2 infection 227 days before.

© 2022 Eythorsson E et al. *JAMA Network Open.*

eAppendix 2. Statistical Code

Libraries and session info

```
library(tidyverse)
library(readxl)
library(lubridate)
library(survminer)
library(rms)
library(knitr)

version
sessionInfo()
```

Import and data cleaning

Here we import a previously cleaned dataset for all administered vaccine doses against SARS-CoV-2 in Iceland till 2021-01-10

```
df_vacc <- read_csv("_output/df_vacc_wide_2022-01-19.csv")
```

PCR results are contained in several different data files as they were recieved over time. ### First PCR dataset The first file contains the results of all PCR tests done between 2020-02-01 (the first PCR test for SARS-CoV-2 in Iceland) and 2021-01-26

```
df_pcr_20210126 <- read_excel("_data/Veiruniðurstöður.xlsx")

df_pcr_20210126 <- df_pcr_20210126 %>%
  rename(
    kt = KENNITALA,
    sex = KYN,
    age = ALDUR,
    zip = POSTNR,
    date_death = DOD,
    distr = LANDSHLUTI
  ) %>%
  rename_at(
    .vars = vars(starts_with("RANNSÓKN")),
    .funs = ~ str_replace(., "RANNSÓKN", "date")
  ) %>%
  rename_at(
    .vars = vars(starts_with("NIDURSTADA")),
    .funs = ~ str_replace(., "NIDURSTADA", "result")
  ) %>%
  rename_at(
    .vars = vars(starts_with("SKRAD_NIDURSTADA")),
    .funs = ~ str_replace(., "SKRAD_NIDURSTADA", "type")
  )

df_pcr_20210126 <- df_pcr_20210126 %>%
```

© 2022 Eythorsson E et al. *JAMA Network Open.*

```
  pivot_longer(
    cols = matches("_[0-9]+$"),
    names_pattern = "(.*)_([0-9]+)",
    names_to = c(".value", "test_nr")
  ) %>%
  filter_at(
    vars(date:result),
    any_vars(!is.na(.))
  )
```

Now because we went from wide to long, and in many cases the age of the patient has changed, we have to recalculate the age and then pass a filter for distinct rows.

```
df_pcr_20210126 <- df_pcr_20210126 %>%
  mutate(
    birth_d = str_sub(.$kt, 1, 2),
    birth_m =  str_sub(.$kt, 3, 4),
    birth_y = case_when(
      str_sub(.$kt, 10, 10) == 0 ~ paste0(20, str_sub(.$kt, 5, 6)),
      str_sub(.$kt, 10, 10) == 9 ~ paste0(19, str_sub(.$kt, 5, 6)),
      str_sub(.$kt, 10, 10) == 8 ~ paste0(18, str_sub(.$kt, 5, 6))
    )
  )  %>%
  mutate(birth_date = as.Date(paste(.$birth_y, .$birth_m, .$birth_d, sep = "-
"))) %>%
  mutate(age = floor(as.numeric(difftime(date, birth_date, units = "days"))/3
65.25)) %>%
  mutate(date_death = as.Date(date_death, format = "%d.%m.%Y")) %>%
  distinct()
```

Two national identification numbers were incorrect giving an impossible age. These were removed.

```
df_pcr_20210126 <- df_pcr_20210126 %>%
  filter(age <= 104)
```

Here we simplify the categorizations for sex and result of PCR tests.

```
df_pcr_20210126 <- df_pcr_20210126 %>%
  mutate(
    sex = case_when(
      sex == "Karl" ~ "Male",
      sex == "Drengur" ~ "Male",
      sex == "Kona" ~ "Female",
      sex == "Stúlka" ~ "Female",
      TRUE ~ NA_character_
    ),
    date = as.Date(date),
    result = case_when(
      type == "Covid19Negative" ~ "negative",
      type == "COVID-19 (SARS-CoV-2) nucleic acid test: Negativ" ~ "negative"
```

© 2022 Eythorsson E et al. *JAMA Network Open.*

```
,
      type == "COVID-19 (SARS-CoV-2) nucleic acid test: Negative" ~ "negative
",
      type == "Covid19Positive" ~ "positive",
      type == "Covid19Positive-Einkenni" ~ "positive",
      type == "Covid19Positive-Ferðamaður" ~ "positive",
      type == "Covid19Positive-Landamæri" ~ "positive",
      type == "Covid19Positive-Rakning" ~ "positive",
      type == "Covid19Positive-Skimun" ~ "positive",
      type == "Ekkert túlkunarskilyrði uppfyllt" ~ NA_character_,
      type == "Inconclusive" ~ NA_character_,
      type == "Inconclusive-Einkenni" ~ NA_character_,
      type == "Inconclusive-Ferðamaður" ~ NA_character_,
      type == "Inconclusive-Landamæri" ~ NA_character_,
      type == "Inconclusive-Rakning" ~ NA_character_,
      type == "Inconclusive-Skimun" ~ NA_character_,
      type == "Ekki gert" ~ NA_character_,
      type == "Inconclusive" ~ NA_character_,
      type == "Jákvæð" ~ "positive",
      type == "Lág-jákvæð" ~ "positive",
      type == "Neikvæð" ~ "negative",
      type == "Of lítið sýni til mælinga." ~ NA_character_,
      type == "Vafasvar" ~ NA_character_
    )
  )

df_pcr_20210126 <- df_pcr_20210126 %>%
  select(kt, sex, age, date, result, date_death, distr)
```

Second PCR dataset

The second file contains a slightly overlapping dataset of results of all PCR tests done between 2021-01-01 and 2021-08-09.

```
df_pcr_20210809 <-
  read_excel("_data/Covid-rannsókn/Covid_veirulisti.xlsx")

df_pcr_20210809 <- df_pcr_20210809 %>%
  rename(
    kt = Kennitala,
    sex = KYN,
    age = ALDUR,
    zip = POSTNR,
    date_death = DOD,
    distr = LANDSHLUTI
  ) %>%
  rename_at(
    .vars = vars(starts_with("RANNSÓKN")),
    .funs = ~ str_replace(., "RANNSÓKN", "date")
  ) %>%
```

© 2022 Eythorsson E et al. *JAMA Network Open.*

```
  rename_at(
    .vars = vars(starts_with("NIDURSTADA")),
    .funs = ~ str_replace(., "NIDURSTADA", "result")
  ) %>%
  rename_at(
    .vars = vars(starts_with("SKRAD_NIDURSTADA")),
    .funs = ~ str_replace(., "SKRAD_NIDURSTADA", "type")
  )

df_pcr_20210809 <- df_pcr_20210809 %>%
  pivot_longer(
    cols = matches("_[0-9]+$"),
    names_pattern = "(.*)_([0-9]+)",
    names_to = c(".value", "test_nr")
  ) %>%
  filter_at(vars(date:result),
            any_vars(!is.na(.)))
```

Now because we went from wide to long, and in many cases the age of the patient has changed, we have to recalculate the age and then pass a filter for distinct rows.

```
df_pcr_20210809 <- df_pcr_20210809 %>%
  mutate(
    birth_d = str_sub(.$kt, 1, 2),
    birth_m =  str_sub(.$kt, 3, 4),
    birth_y = case_when(
      str_sub(.$kt, 10, 10) == 0 ~ paste0(20, str_sub(.$kt, 5, 6)),
      str_sub(.$kt, 10, 10) == 9 ~ paste0(19, str_sub(.$kt, 5, 6)),
      str_sub(.$kt, 10, 10) == 8 ~ paste0(18, str_sub(.$kt, 5, 6))
    )
  )  %>%
  mutate(birth_date = as.Date(paste(.$birth_y, .$birth_m, .$birth_d, sep = "-
"))) %>%
  mutate(age = floor(as.numeric(difftime(date, birth_date, units = "days"))/3
65.25)) %>%
  mutate(date_death = as.Date(date_death, format = "%d.%m.%Y")) %>%
  distinct()
```

Several national identification numbers were incorrectly coded giving impossible age values, these were removed.

```
df_pcr_20210809 <- df_pcr_20210809 %>%
  filter(age >= 0, age <= 108)
```

Here we simplify the categorizations for sex and result of PCR tests.

```
df_pcr_20210809<- df_pcr_20210809 %>%
  mutate(
    sex = case_when(
      sex == "Karl" ~ "Male",
      sex == "Drengur" ~ "Male",
```

```
      sex == "Kona" ~ "Female",
      sex == "Stúlka" ~ "Female",
      TRUE ~ NA_character_
    ),
    date = as.Date(date),
    result = case_when(
      type == "Covid19Negative" ~ "negative",
      type == "COVID-19 (SARS-CoV-2) nucleic acid test: Negativ" ~ "negative"
,
      type == "COVID-19 (SARS-CoV-2) nucleic acid test: Negative" ~ "negative
",
      type == "Covid19Positive" ~ "positive",
      type == "Covid19Positive-Einkenni" ~ "positive",
      type == "Covid19Positive-Ferðamaður" ~ "positive",
      type == "Covid19Positive-Landamæri" ~ "positive",
      type == "Covid19Positive-Rakning" ~ "positive",
      type == "Covid19Positive-Skimun" ~ "positive",
      type == "Ekkert túlkunarskilyrði uppfyllt" ~ NA_character_,
      type == "Inconclusive" ~ NA_character_,
      type == "Inconclusive-Einkenni" ~ NA_character_,
      type == "Inconclusive-Ferðamaður" ~ NA_character_,
      type == "Inconclusive-Landamæri" ~ NA_character_,
      type == "Inconclusive-Rakning" ~ NA_character_,
      type == "Inconclusive-Skimun" ~ NA_character_,
      type == "Ekki gert" ~ NA_character_,
      type == "Inconclusive" ~ NA_character_,
      type == "Jákvæð" ~ "positive",
      type == "Lág-jákvæð" ~ "positive",
      type == "Neikvæð" ~ "negative",
      type == "Of lítið sýni til mælinga." ~ NA_character_,
      type == "Vafasvar" ~ NA_character_
    )
  )

df_pcr_20210809 <- df_pcr_20210809 %>%
  select(kt, sex, age, date, result, date_death2 = date_death)
```

Third PCR dataset

The third dataset contains all PCR test results from 2021-07-15 to 2021-09-02.

```
df_pcr_agust <- read_excel(
  "_data/Veirurannsóknir niðurstöður agust 2021.xlsx",
  col_types = c("text", "text", "numeric", "text", "text", "text", "text",
                "text", "text", "text", "text", "text", "text", "text", "text
",
                "text", "text", "text", "text", "text", "text", "text", "text
",
                "text", "text", "text", "text", "text", "text", "text",
                "text", "text", "text", "text", "text", "text",
```

© 2022 Eythorsson E et al. *JAMA Network Open.*

```
                "text", "text", "text", "text", "text", "text",
                "text", "text", "text", "text", "text", "text",
                "text", "text", "text", "text", "text", "text",
                "text", "text", "text", "text", "text", "text",
                "text", "text", "text", "text", "text", "text")
)

df_pcr_agust <- df_pcr_agust %>%
  rename(
    kt = KENNITALA,
    sex = KYN,
    age = ALDUR,
    zip = POSTNR,
    date_death = DOD,
    distr = LANDSHLUTI
  ) %>%
  rename_at(
    .vars = vars(starts_with("RANNSÓKN")),
    .funs = ~ str_replace(., "RANNSÓKN", "date")
  ) %>%
  rename_at(
    .vars = vars(starts_with("NIDURSTADA")),
    .funs = ~ str_replace(., "NIDURSTADA", "result")
  ) %>%
  rename_at(
    .vars = vars(starts_with("SKRAD_NIDURSTADA")),
    .funs = ~ str_replace(., "SKRAD_NIDURSTADA", "type")
  )

df_pcr_agust <- df_pcr_agust %>%
  pivot_longer(
    cols = matches("_[0-9]+$"),
    names_pattern = "(.*)_([0-9]+)",
    names_to = c(".value", "test_nr")
  ) %>%
  filter_at(vars(date:result),
            any_vars(!is.na(.)))
```

Date is now in Excel Mac format, this must be changed.

```
df_pcr_agust <- df_pcr_agust %>%
  mutate(
    date = as.Date(floor(as.numeric(date)), origin = as.Date("1900-01-01")),
    date_death = as.Date(floor(as.numeric(date_death)), origin = as.Date("190
0-01-01"))) %>%
  filter(!is.na(date))
```

Now because we went from wide to long, and in many cases the age of the patient has changed, we have to recalculate the age and then pass a filter for distinct rows.

```
df_pcr_agust <- df_pcr_agust %>%
  mutate(
    birth_d = str_sub(.$kt, 1, 2),
    birth_m =  str_sub(.$kt, 3, 4),
    birth_y = case_when(
      str_sub(.$kt, 10, 10) == 0 ~ paste0(20, str_sub(.$kt, 5, 6)),
      str_sub(.$kt, 10, 10) == 9 ~ paste0(19, str_sub(.$kt, 5, 6)),
      str_sub(.$kt, 10, 10) == 8 ~ paste0(18, str_sub(.$kt, 5, 6))
    )
  )  %>%
  mutate(birth_date = as.Date(paste(.$birth_y, .$birth_m, .$birth_d, sep = "-
"))) %>%
  mutate(age = floor(as.numeric(difftime(date, birth_date, units = "days"))/3
65.25)) %>%
  distinct()
```

Several impossible age values were removed.

```
df_pcr_agust <- df_pcr_agust %>%
  filter(age >= 0, age <= 104)
```

Here we simplify the categorizations for sex and result of PCR tests.

```
df_pcr_agust<- df_pcr_agust %>%
  mutate(
    sex = case_when(
      sex == "Karl" ~ "Male",
      sex == "Drengur" ~ "Male",
      sex == "Kona" ~ "Female",
      sex == "Stúlka" ~ "Female",
      TRUE ~ NA_character_
    ),
    date = as.Date(date),
    result = case_when(
      type == "Covid19Negative" ~ "negative",
      type == "COVID-19 (SARS-CoV-2) nucleic acid test: Negativ" ~ "negative"
,
      type == "COVID-19 (SARS-CoV-2) nucleic acid test: Negative" ~ "negative
",
      type == "Covid19Positive" ~ "positive",
      type == "Covid19Positive-Einkenni" ~ "positive",
      type == "Covid19Positive-Ferðamaður" ~ "positive",
      type == "Covid19Positive-Landamæri" ~ "positive",
      type == "Covid19Positive-Rakning" ~ "positive",
      type == "Covid19Positive-Skimun" ~ "positive",
      type == "Ekkert túlkunarskilyrði uppfyllt" ~ NA_character_,
      type == "Inconclusive" ~ NA_character_,
      type == "Inconclusive-Einkenni" ~ NA_character_,
      type == "Inconclusive-Ferðamaður" ~ NA_character_,
      type == "Inconclusive-Landamæri" ~ NA_character_,
      type == "Inconclusive-Rakning" ~ NA_character_,
```

© 2022 Eythorsson E et al. *JAMA Network Open.*

```
      type == "Inconclusive-Skimun" ~ NA_character_,
      type == "Ekki gert" ~ NA_character_,
      type == "Inconclusive" ~ NA_character_,
      type == "Jákvæð" ~ "positive",
      type == "Lág-jákvæð" ~ "positive",
      type == "Neikvæð" ~ "negative",
      type == "Of lítið sýni til mælinga." ~ NA_character_,
      type == "Vafasvar" ~ NA_character_
    )
  )

df_pcr_agust <- df_pcr_agust %>%
  select(kt, sex, age, date, result, date_death3 = date_death)
```

Fourth PCR dataset

The fourth dataset contains all PCR test results from 2021-09-03 to 2022-01-14.

```
df_pcr_20220114 <- read_excel(
  "_data/Niðurstöður janúar 2022/Veirurannsóknir niðurstöður.xlsx",
  col_types = c("text", "text", "numeric", "text", "text", "text", "text",
                "text", "text", "text", "text", "text", "text", "text", "text
",
                "text", "text", "text", "text", "text", "text", "text", "text
",
                "text", "text", "text", "text", "text", "text", "text", "text
",
                "text", "text", "text", "text", "text", "text", "text", "text
",
                "text", "text", "text", "text", "text", "text", "text", "text
",
                "text", "text", "text", "text", "text", "text", "text", "text
",
                "text", "text", "text", "text", "text", "text", "text", "text
",
                "text", "text", "text", "text", "text", "text", "text", "text
",
                "text", "text", "text", "text", "text", "text", "text", "text
",
                "text", "text", "text", "text", "text", "text", "text", "text
",
                "text", "text", "text", "text", "text", "text", "text", "text
",
                "text", "text", "text", "text", "text", "text", "text", "text
",
                "text", "text", "text", "text", "text", "text", "text", "text
",
                "text", "text", "text", "text", "text", "text", "text", "text
",
                "text", "text", "text", "text", "text", "text", "text", "text
```

© 2022 Eythorsson E et al. *JAMA Network Open.*

```
",
                "text", "text", "text", "text", "text"
  ))

df_pcr_20220114 <- df_pcr_20220114 %>%
  rename(
    kt = KENNITALA,
    sex = KYN,
    age = ALDUR,
    zip = POSTNR,
    date_death = DOD,
    distr = LANDSHLUTI
  ) %>%
  rename_at(
    .vars = vars(starts_with("RANNSÓKN")),
    .funs = ~ str_replace(., "RANNSÓKN", "date")
  ) %>%
  rename_at(
    .vars = vars(starts_with("NIDURSTADA")),
    .funs = ~ str_replace(., "NIDURSTADA", "result")
  ) %>%
  rename_at(
    .vars = vars(starts_with("SKRAD_NIDURSTADA")),
    .funs = ~ str_replace(., "SKRAD_NIDURSTADA", "type")
  )

df_pcr_20220114 <- df_pcr_20220114 %>%
  pivot_longer(
    cols = matches("_[0-9]+$"),
    names_pattern = "(.*)_([0-9]+)",
    names_to = c(".value", "test_nr")
  ) %>%
  filter_at(
    vars(date:result),
    any_vars(!is.na(.))
  )
```

Date is now in Excel Mac format, this must be changed.

```
df_pcr_20220114 <- df_pcr_20220114 %>%
  mutate(
    date = as.Date(floor(as.numeric(date)), origin = as.Date("1900-01-01")),
    date_death = as.Date(floor(as.numeric(date_death)), origin = as.Date("190
0-01-01"))) %>%
  filter(!is.na(date))
```

Now because we went from wide to long, and in many cases the age of the patient has changed, we have to recalculate the age and then pass a filter for distinct rows.

```
df_pcr_20220114 <- df_pcr_20220114 %>%
  mutate(
    birth_d = str_sub(.$kt, 1, 2),
    birth_m =  str_sub(.$kt, 3, 4),
    birth_y = case_when(
      str_sub(.$kt, 10, 10) == 0 ~ paste0(20, str_sub(.$kt, 5, 6)),
      str_sub(.$kt, 10, 10) == 9 ~ paste0(19, str_sub(.$kt, 5, 6)),
      str_sub(.$kt, 10, 10) == 8 ~ paste0(18, str_sub(.$kt, 5, 6))
    )
  )  %>%
  mutate(birth_date = as.Date(paste(.$birth_y, .$birth_m, .$birth_d, sep = "-
"))) %>%
  mutate(age = floor(as.numeric(difftime(date, birth_date, units = "days"))/3
65.25)) %>%
  distinct()
```

Several impossible age values were removed.

```
df_pcr_20220114 <- df_pcr_20220114 %>%
  filter(age >= 0, age <= 108)
```

Here we simplify the categorizations for sex and result of PCR tests.

```
df_pcr_20220114<- df_pcr_20220114 %>%
  mutate(
    sex = case_when(
      sex == "Karl" ~ "Male",
      sex == "Drengur" ~ "Male",
      sex == "Kona" ~ "Female",
      sex == "Stúlka" ~ "Female",
      TRUE ~ NA_character_
    ),
    date = as.Date(date),
    result = case_when(
      type == "Covid19Negative" ~ "negative",
      type == "COVID-19 (SARS-CoV-2) nucleic acid test: Negativ" ~ "negative"
,
      type == "COVID-19 (SARS-CoV-2) nucleic acid test: Negative" ~ "negative
",
      type == "Covid19Positive" ~ "positive",
      type == "Covid19Positive-Einkenni" ~ "positive",
      type == "Covid19Positive-Ferðamaður" ~ "positive",
      type == "Covid19Positive-Landamæri" ~ "positive",
      type == "Covid19Positive-Rakning" ~ "positive",
      type == "Covid19Positive-Skimun" ~ "positive",
      type == "Ekkert túlkunarskilyrði uppfyllt" ~ NA_character_,
      type == "Inconclusive" ~ NA_character_,
      type == "Inconclusive-Einkenni" ~ NA_character_,
      type == "Inconclusive-Ferðamaður" ~ NA_character_,
      type == "Inconclusive-Landamæri" ~ NA_character_,
      type == "Inconclusive-Rakning" ~ NA_character_,
```

© 2022 Eythorsson E et al. *JAMA Network Open.*

```
      type == "Inconclusive-Skimun" ~ NA_character_,
      type == "Ekki gert" ~ NA_character_,
      type == "Inconclusive" ~ NA_character_,
      type == "Jákvæð" ~ "positive",
      type == "Lág-jákvæð" ~ "positive",
      type == "Neikvæð" ~ "negative",
      type == "Of lítið sýni til mælinga." ~ NA_character_,
      type == "Vafasvar" ~ NA_character_
    )
  )

df_pcr_20220114 <- df_pcr_20220114 %>%
  select(kt, sex, age, date, result, date_death4 = date_death)
```

Fifth PCR dataset

The fifth dataset contains an overlapping dataset of all PCR test results from 2021-12-17 to 2022-01-26.

```
df_pcr_20220126 <- read_excel("_data/Niðurstöður janúar 2022 viðbot/Veirurann
sóknir niðurstöður.xlsx",
                             col_types = c("text", "text", "numeric",
                                           "text", "text", "text", "text",
                                           "text", "text", "text", "text",
                                           "text", "text", "text", "text",
                                           "text", "text", "text", "text",
                                           "text", "text", "text", "text",
                                           "text", "text", "text", "text",
                                           "text", "text", "text", "text",
                                           "text", "text", "text",
                                           "text", "text", "text",
                                           "text", "text", "text",
                                           "text", "text", "text",
                                           "text", "text", "text",
                                           "text", "text", "text",
                                           "text", "text", "text",
                                           "text", "text", "text",
                                           "text", "text", "text",
                                           "text", "text", "text",
                                           "text", "text", "text",
                                           "text", "text"))

df_pcr_20220126 <- df_pcr_20220126 %>%
  rename(
    kt = KENNITALA,
    sex = KYN,
    age = ALDUR,
    zip = POSTNR,
    date_death = DOD,
    distr = LANDSHLUTI
```

© 2022 Eythorsson E et al. *JAMA Network Open.*

```
  ) %>%
  rename_at(
    .vars = vars(starts_with("RANNSÓKN")),
    .funs = ~ str_replace(., "RANNSÓKN", "date")
  ) %>%
  rename_at(
    .vars = vars(starts_with("NIDURSTADA")),
    .funs = ~ str_replace(., "NIDURSTADA", "result")
  ) %>%
  rename_at(
    .vars = vars(starts_with("SKRAD_NIDURSTADA")),
    .funs = ~ str_replace(., "SKRAD_NIDURSTADA", "type")
  )

df_pcr_20220126 <- df_pcr_20220126 %>%
  pivot_longer(
    cols = matches("_[0-9]+$"),
    names_pattern = "(.*)_([0-9]+)",
    names_to = c(".value", "test_nr")
  ) %>%
  filter_at(
    vars(date:result),
    any_vars(!is.na(.))
  )
```

Date is now in Excel Mac format, this must be changed.

```
df_pcr_20220126 <- df_pcr_20220126 %>%
  mutate(
    date = as.Date(floor(as.numeric(date)), origin = as.Date("1900-01-01")),
    date_death = as.Date(floor(as.numeric(date_death)), origin = as.Date("190
0-01-01"))) %>%
  filter(!is.na(date))
```

Now because we went from wide to long, and in many cases the age of the patient has changed, we have to recalculate the age and then pass a filter for distinct rows.

```
df_pcr_20220126 <- df_pcr_20220126 %>%
  mutate(
    birth_d = str_sub(.$kt, 1, 2),
    birth_m =  str_sub(.$kt, 3, 4),
    birth_y = case_when(
      str_sub(.$kt, 10, 10) == 0 ~ paste0(20, str_sub(.$kt, 5, 6)),
      str_sub(.$kt, 10, 10) == 9 ~ paste0(19, str_sub(.$kt, 5, 6)),
      str_sub(.$kt, 10, 10) == 8 ~ paste0(18, str_sub(.$kt, 5, 6))
    )
  )  %>%
  mutate(birth_date = as.Date(paste(.$birth_y, .$birth_m, .$birth_d, sep = "-
"))) %>%
  mutate(age = floor(as.numeric(difftime(date, birth_date, units = "days"))/3
```

© 2022 Eythorsson E et al. *JAMA Network Open.*

```r
65.25)) %>%
  distinct()
```

Several impossible age values were removed.

```r
df_pcr_20220126 <- df_pcr_20220126 %>%
  filter(age >= 0, age <= 108)
```

Here we simplify the categorizations for sex and result of PCR tests.

```r
df_pcr_20220126<- df_pcr_20220126 %>%
  mutate(
    sex = case_when(
      sex == "Karl" ~ "Male",
      sex == "Drengur" ~ "Male",
      sex == "Kona" ~ "Female",
      sex == "Stúlka" ~ "Female",
      TRUE ~ NA_character_
    ),
    date = as.Date(date),
    result = case_when(
      type == "Covid19Negative" ~ "negative",
      type == "COVID-19 (SARS-CoV-2) nucleic acid test: Negativ" ~ "negative"
,
      type == "COVID-19 (SARS-CoV-2) nucleic acid test: Negative" ~ "negative
",
      type == "Covid19Positive" ~ "positive",
      type == "Covid19Positive-Einkenni" ~ "positive",
      type == "Covid19Positive-Ferðamaður" ~ "positive",
      type == "Covid19Positive-Landamæri" ~ "positive",
      type == "Covid19Positive-Rakning" ~ "positive",
      type == "Covid19Positive-Skimun" ~ "positive",
      type == "Ekkert túlkunarskilyrði uppfyllt" ~ NA_character_,
      type == "Inconclusive" ~ NA_character_,
      type == "Inconclusive-Einkenni" ~ NA_character_,
      type == "Inconclusive-Ferðamaður" ~ NA_character_,
      type == "Inconclusive-Landamæri" ~ NA_character_,
      type == "Inconclusive-Rakning" ~ NA_character_,
      type == "Inconclusive-Skimun" ~ NA_character_,
      type == "Ekki gert" ~ NA_character_,
      type == "Inconclusive" ~ NA_character_,
      type == "Jákvæð" ~ "positive",
      type == "Lág-jákvæð" ~ "positive",
      type == "Neikvæð" ~ "negative",
      type == "Of lítið sýni til mælinga." ~ NA_character_,
      type == "Vafasvar" ~ NA_character_
    )
  )

df_pcr_20220126 <- df_pcr_20220126 %>%
  select(kt, sex, age, date, result, date_death5 = date_death)
```

© 2022 Eythorsson E et al. *JAMA Network Open.*

Sixth PCR dataset

The sixth dataset contains an overlapping dataset of all PCR test results from 2021-12-17 to 2022-01-26.

```
df_pcr_20220214 <- read_excel(
  "_data/Niðurstöður febrúar 2022/Veirurannsóknir niðurstöður.xlsx",
  col_types = c("text", "text", "numeric", "text", "text", "text", "text",
                "text", "text", "text", "text", "text", "text", "text", "text
",
                "text", "text", "text", "text", "text", "text", "text", "text
",
                "text", "text", "text", "text", "text", "text", "text", "text
",
                "text", "text", "text", "text", "text", "text", "text", "text
", "text",
                "text", "text", "text", "text", "text", "text", "text", "text
", "text",
                "text", "text", "text", "text", "text", "text", "text", "text
", "text",
                "text", "text", "text", "text", "text", "text", "text", "text
",
                "text", "text", "text", "text", "text", "text", "text", "text
",
                "text", "text", "text", "text", "text", "text", "text"
  ))

df_pcr_20220214 <- df_pcr_20220214 %>%
  rename(
    kt = KENNITALA,
    sex = KYN,
    age = ALDUR,
    zip = POSTNR,
    date_death = DOD,
    distr = LANDSHLUTI
  ) %>%
  rename_at(
    .vars = vars(starts_with("RANNSÓKN")),
    .funs = ~ str_replace(., "RANNSÓKN", "date")
  ) %>%
  rename_at(
    .vars = vars(starts_with("NIDURSTADA")),
    .funs = ~ str_replace(., "NIDURSTADA", "result")
  ) %>%
  rename_at(
    .vars = vars(starts_with("SKRAD_NIDURSTADA")),
    .funs = ~ str_replace(., "SKRAD_NIDURSTADA", "type")
  )

df_pcr_20220214 <- df_pcr_20220214 %>%
```

© 2022 Eythorsson E et al. *JAMA Network Open.*

```
  pivot_longer(
    cols = matches("_[0-9]+$"),
    names_pattern = "(.*)_([0-9]+)",
    names_to = c(".value", "test_nr")
  ) %>%
  filter_at(
    vars(date:result),
    any_vars(!is.na(.))
  )
```

Date is now in Excel Mac format, this must be changed.

```
df_pcr_20220214 <- df_pcr_20220214 %>%
  mutate(
    date = as.Date(floor(as.numeric(date)), origin = as.Date("1900-01-01")),
    date_death = as.Date(floor(as.numeric(date_death)), origin = as.Date("190
0-01-01"))) %>%
  filter(!is.na(date))
```

Now because we went from wide to long, and in many cases the age of the patient has changed, we have to recalculate the age and then pass a filter for distinct rows.

```
df_pcr_20220214 <- df_pcr_20220214 %>%
  mutate(
    birth_d = str_sub(.$kt, 1, 2),
    birth_m =  str_sub(.$kt, 3, 4),
    birth_y = case_when(
      str_sub(.$kt, 10, 10) == 0 ~ paste0(20, str_sub(.$kt, 5, 6)),
      str_sub(.$kt, 10, 10) == 9 ~ paste0(19, str_sub(.$kt, 5, 6)),
      str_sub(.$kt, 10, 10) == 8 ~ paste0(18, str_sub(.$kt, 5, 6))
    )
  )  %>%
  mutate(birth_date = as.Date(paste(.$birth_y, .$birth_m, .$birth_d, sep = "-
"))) %>%
  mutate(age = floor(as.numeric(difftime(date, birth_date, units = "days"))/3
65.25)) %>%
  distinct()
```

Several impossible age values were removed.

```
df_pcr_20220214 <- df_pcr_20220214 %>%
  filter(age >= 0, age <= 108)
```

Here we simplify the categorizations for sex and result of PCR tests.

```
df_pcr_20220214 <- df_pcr_20220214 %>%
  mutate(
    sex = case_when(
      sex == "Karl" ~ "Male",
      sex == "Drengur" ~ "Male",
      sex == "Kona" ~ "Female",
```

© 2022 Eythorsson E et al. *JAMA Network Open.*

```
      sex == "Stúlka" ~ "Female",
      TRUE ~ NA_character_
    ),
    date = as.Date(date),
    result = case_when(
      type == "Covid19Negative" ~ "negative",
      type == "COVID-19 (SARS-CoV-2) nucleic acid test: Negativ" ~ "negative"
,
      type == "COVID-19 (SARS-CoV-2) nucleic acid test: Negative" ~ "negative
",
      type == "Covid19Positive" ~ "positive",
      type == "Covid19Positive-Einkenni" ~ "positive",
      type == "Covid19Positive-Ferðamaður" ~ "positive",
      type == "Covid19Positive-Landamæri" ~ "positive",
      type == "Covid19Positive-Rakning" ~ "positive",
      type == "Covid19Positive-Skimun" ~ "positive",
      type == "Ekkert túlkunarskilyrði uppfyllt" ~ NA_character_,
      type == "Inconclusive" ~ NA_character_,
      type == "Inconclusive-Einkenni" ~ NA_character_,
      type == "Inconclusive-Ferðamaður" ~ NA_character_,
      type == "Inconclusive-Landamæri" ~ NA_character_,
      type == "Inconclusive-Rakning" ~ NA_character_,
      type == "Inconclusive-Skimun" ~ NA_character_,
      type == "Ekki gert" ~ NA_character_,
      type == "Inconclusive" ~ NA_character_,
      type == "Jákvæð" ~ "positive",
      type == "Lág-jákvæð" ~ "positive",
      type == "Neikvæð" ~ "negative",
      type == "Of lítið sýni til mælinga." ~ NA_character_,
      type == "Vafasvar" ~ NA_character_
    )
  )

df_pcr_20220214 <- df_pcr_20220214 %>%
  select(kt, sex, age, date, result, date_death6 = date_death)
```

Combining the PCR datasets

```
df_pcr <- df_pcr_20210126 %>%
  union_all(df_pcr_20210809) %>%
  union_all(df_pcr_agust) %>%
  union_all(df_pcr_20220114) %>%
  union_all(df_pcr_20220126) %>%
  union_all(df_pcr_20220214) %>%

  #' This code is necessary to overwrite NA values for date of death in earli
er
  #' datasets with known value when a date of death has later been logged
  mutate(
    date_death = case_when(
      !is.na(date_death) ~ date_death,
```

© 2022 Eythorsson E et al. *JAMA Network Open.*

```
      is.na(date_death) & !is.na(date_death2) ~ date_death2,
      is.na(date_death) & is.na(date_death2) & !is.na(date_death3) ~ date_dea
th3,
      is.na(date_death) & is.na(date_death2) & is.na(date_death3) &
        !is.na(date_death4) ~ date_death4,
      is.na(date_death) & is.na(date_death2) & is.na(date_death3) &
        is.na(date_death4) & !is.na(date_death5) ~ date_death5,
      is.na(date_death) & is.na(date_death2) & is.na(date_death3) &
        is.na(date_death4) & is.na(date_death5) & !is.na(date_death6) ~ date_
death6,
      TRUE ~ date_death6
    )
  ) %>%
  select(kt, sex, age, date, result, date_death) %>%
  distinct()

df_pcr <- df_pcr %>%
  group_by(kt) %>%
  mutate(test_nr = row_number()) %>%
  ungroup()
```

Included were the results of PCR tests that had arrived before 16:00 on 2022-02-14. As this was not a full day we removed these observations from the dataset.

```
df_pcr <- df_pcr %>%
  filter(date <= as.Date("2022-02-13"))
```

Because the data is in long format, the date of death is still missing for negative PCR results occurring before the date of death was known. This code spreads the known date of death across all rows belonging to the same individual.

```
df_pcr <- df_pcr %>%
  group_by(kt) %>%
  fill(date_death, .direction = "downup") %>%
  mutate(date_death = max(date_death)) %>%
  ungroup()
```

Main analysis

Based on 30-day reinfection definition (sensitivity analysis)

First, we define a reinfection to have occurred if a PCR becomes positive 30 days or longer from a previously positive PCR.

```
df_covid_30 <- df_pcr %>%
  filter(result == "positive") %>%
  group_by(kt) %>%
  arrange(kt, date) %>%
  mutate(
    test_nr = row_number(),
    t = as.numeric(difftime(date, lag(date), units = "days")),
```

© 2022 Eythorsson E et al. *JAMA Network Open.*

```
    t = if_else(is.na(t), 0, t),
    cumsum = cumsum(t)) %>%
  ungroup() %>%
  filter(test_nr == 1 | t >= 30) %>%
  mutate(reinfected = if_else(test_nr > 1, 1, 0)) %>%
  left_join(
    df_pcr %>%
      select(kt, date_death) %>%
      distinct()
  )

df_covid_30 <- df_covid_30 %>%
  mutate(age_group = factor(case_when(
    age < 18 ~ "17yr or younger",
    age >= 18 & age <= 29 ~ "18-29yr",
    age >= 30 & age <= 49 ~ "30-49yr",
    age >= 50 & age <= 74 ~ "50-74yr",
    age >= 75 ~ "75yr or older"
  ), levels = c("17yr or younger", "18-29yr", "30-49yr", "50-74yr", "75yr or
older")
  ))
```

We must ensure that each person is only represented in one line, such that the date column for those who are reinfected represents the date of their first positive PCR test, but the line otherwise refers to their second infection.

```
kt_reinfected_30 <- df_covid_30 %>%
  filter(reinfected == 1) %>%
  pull(kt)

df_surv_30 <- df_covid_30

df_surv_30 <- df_surv_30 %>%
  mutate(
    remove = case_when(
      kt %in% kt_reinfected_30 & reinfected == 0 ~ "remove",
      TRUE ~ "dont_remove"
    )
  ) %>%
  filter(remove != "remove")

df_surv_30 <- df_surv_30 %>%
  mutate(date_censor = case_when(
    reinfected == 1 ~ date,
    date_death <= as.Date("2022-02-13") ~ date_death,
    TRUE ~ as.Date("2022-02-13")
  )) %>%
  left_join(
    df_covid_30 %>%
      filter(kt %in% kt_reinfected_30, reinfected == 0) %>%
```

© 2022 Eythorsson E et al. *JAMA Network Open.*

```
      select(kt, date_fix = date)
  ) %>%
  mutate(date = if_else(reinfected == 1, date_fix, date)) %>%
  mutate(
    date_atrisk = date + 30,
    surv_time = as.numeric(difftime(date_censor, as.Date("2021-12-01"), units
= "days"))
  ) %>%
  filter(date_atrisk <= date_censor)
```

Here we add the vaccination data. Because so few individuals had received a booster shot by December 1, 2021 had become reinfected, we dichotomized vaccine status to ≤1 dose and ≥2 doses.

```
df_surv_30 <- df_surv_30 %>%
  mutate(
    time_to_dec1 = as.numeric(difftime(as.Date("2021-12-01"), date, units = "
days"))) %>%
  left_join(
    df_vacc %>%
      select(-starts_with("age")) %>%
      mutate(across(.cols = starts_with("date"), .fns = ~as.Date(.x)))
  ) %>%
  mutate(vacc_status = factor(case_when(
    !is.na(date_4) & date_4 <= as.Date("2021-12-01") ~ "Two or more vaccine d
oses",
    !is.na(date_3) & date_3 + 14 <= as.Date("2021-12-01") ~ "Two or more vacc
ine doses",
    !is.na(date_3) & date_3 <= as.Date("2021-12-01") & date_3 + 14 >= as.Date
("2021-12-01") ~ "Two or more vaccine doses",
    !is.na(date_2) & date_2 + 14 <= as.Date("2021-12-01") ~ "Two or more vacc
ine doses",
    !is.na(date_2) & date_2 <= as.Date("2021-12-01") & date_2 + 14 >= as.Date
("2021-12-01") ~ "One dose or unvaccinated",
    !is.na(date_1) & date_1 + 14 <= as.Date("2021-12-01") ~ "One dose or unva
ccinated",
    !is.na(date_1) & date_1 <= as.Date("2021-12-01") & date_1 + 14 >= as.Date
("2021-12-01") ~ "One dose or unvaccinated",
    TRUE ~ "One dose or unvaccinated"
  ), levels = c("One dose or unvaccinated", "Two or more vaccine doses")
  )) %>%
  select(-date_4, -date_3, -date_2, -date_1, -name_4, -name_3, -name_2, -name
_1)
```

Number of patients

Number of patients who had their first positive PCR test at least 30 days before 2021-12-01.

```
df_surv_30 %>%
  filter(
```

© 2022 Eythorsson E et al. *JAMA Network Open.*

```
    date_atrisk <= as.Date("2021-12-01"),
    date_censor >= as.Date("2021-12-01")
  ) %>%
  count(kt) %>%
  nrow()
```

Number of reinfected patients

```
df_surv_30 %>%
  filter(
    date_atrisk <= as.Date("2021-12-01"),
    date_censor >= as.Date("2021-12-01")
  ) %>%
  count(reinfected)
```

Sex by reinfection status

```
df_surv_30 %>%
  filter(
    date_atrisk <= as.Date("2021-12-01"),
    date_censor >= as.Date("2021-12-01")
  ) %>%
  count(reinfected, sex)
```

Distribution of age

```
df_surv_30 %>%
  filter(
    date_atrisk <= as.Date("2021-12-01"),
    date_censor >= as.Date("2021-12-01"),
    reinfected == 0
  ) %>%
  pull(age) %>%
  quantile()

df_surv_30 %>%
  filter(
    date_atrisk <= as.Date("2021-12-01"),
    date_censor >= as.Date("2021-12-01"),
    reinfected == 1
  ) %>%
  pull(age) %>%
  quantile()
```

Time from initial infection

```
df_surv_30 %>%
  filter(
    date_atrisk <= as.Date("2021-12-01"),
    date_censor >= as.Date("2021-12-01")
  ) %>%
  pull(time_to_dec1) %>%
  quantile()
```

© 2022 Eythorsson E et al. *JAMA Network Open.*

Distribution of follow-up times

```
df_surv_30 %>%
  filter(
    date_atrisk <= as.Date("2021-12-01"),
    date_censor >= as.Date("2021-12-01")
  ) %>%
  pull(surv_time) %>%
  quantile()

df_surv_30 %>%
  filter(
    date_atrisk <= as.Date("2021-12-01"),
    date_censor >= as.Date("2021-12-01"),
    reinfected == 1
  ) %>%
  pull(surv_time) %>%
  quantile()
```

Vaccine status by reinfection

```
df_surv_30 %>%
  filter(
    date_atrisk <= as.Date("2021-12-01"),
    date_censor >= as.Date("2021-12-01")
  ) %>%
  count(reinfected, vacc_status)
```

Vaccine status by age group

```
df_surv_30 %>%
  filter(
    date_atrisk <= as.Date("2021-12-01"),
    date_censor >= as.Date("2021-12-01")
  ) %>%
  count(age_group, vacc_status)
```

Table 1 (sensitivity analysis)

```
df_surv_30 %>%
  filter(
    date_atrisk <= as.Date("2021-12-01"),
    date_censor >= as.Date("2021-12-01")
  ) %>%
  count(age_group, reinfected) %>%
  pivot_wider(
    id_cols = age_group,
    names_from = reinfected,
    values_from = n
  ) %>%
  mutate(total = `1`+`0`, prop = `1`/(`1`+`0`)*100) %>%
  select(age_group, total, num_reinfected = `1`, prop) %>%
  kable()
```

© 2022 Eythorsson E et al. *JAMA Network Open.*

Cox regression model

```
covid_datadist <- datadist(
  df_surv_30 %>%
    filter(
      date_censor >= as.Date("2021-12-01"),
      date_atrisk <= as.Date("2021-12-01")
    ) %>%
    select(-date_death))
covid_datadist$limits["Adjust to", "age_group"] <- "18-29yr"
covid_datadist$limits["High:effect", "time_to_dec1"] <- 547
covid_datadist$limits["Low:effect", "time_to_dec1"] <- 91

options(datadist = "covid_datadist")

coxfit_omicron_30 <- cph(Surv(surv_time, reinfected) ~ rcs(time_to_dec1, 5) +
vacc_status + age_group +
                           time_to_dec1 %ia% vacc_status + time_to_dec1 %ia%
age_group + vacc_status * age_group,
                         x = TRUE,
                         y = TRUE,
                         data = df_surv_30 %>%
                           filter(
                             date_censor >= as.Date("2021-12-01"),
                             date_atrisk <= as.Date("2021-12-01")
                           ))

anova(coxfit_omicron_30)

plot(anova(coxfit_omicron_30))

knitr::kable(summary(coxfit_omicron_30))

plot(summary(coxfit_omicron_30))

coxfit_omicron_30 <- cph(Surv(surv_time, reinfected) ~ rcs(time_to_dec1, 5) +
vacc_status + strat(age_group) +
                           time_to_dec1 %ia% vacc_status + time_to_dec1 %ia%
strat(age_group) + vacc_status * strat(age_group),
                         x = TRUE,
                         y = TRUE,
                         data = df_surv_30 %>%
                           filter(
                             date_censor >= as.Date("2021-12-01"),
                             date_atrisk <= as.Date("2021-12-01")
                           ))

survplot_30 <- ggsurvplot(
  fit = survfit(coxfit_omicron_30),
  data = df_surv_30 %>%
    filter(
```

© 2022 Eythorsson E et al. *JAMA Network Open.*

```
      date_censor >= as.Date("2021-12-01"),
      date_atrisk <= as.Date("2021-12-01")
    ),
  fun = "event",
  break.x.by = 15,
  xlim = c(0, 80),
  xlab = "Days from start of Omicron wave (December 1, 2021)",
  ylim = c(0, 0.55),
  ylab = "Cumulative proportion reinfected",
  legend.labs = c(
    "17yr or younger",
    "18-29yr",
    "30-49yr",
    "50-74yr",
    "75yr or older"
  ),
  palette = c(
    "#374E55FF",
    "#DF8F44FF",
    "#00A1D5FF",
    "#B24745FF",
    "#79AF97FF"
    ),
  legend.title = "",
  conf.int = TRUE,
  risk.table = TRUE,
  cumevents = TRUE,
  ggtheme = theme_bw()
)

survplot_30$plot <- survplot_30$plot +
  scale_y_continuous(labels = scales::percent)

survplot_30
```

Logistic regression model

```
fit_omicron_30 <- lrm(
  reinfected ~ rcs(time_to_dec1, 5) + vacc_status + age_group +
    time_to_dec1 %ia% vacc_status + time_to_dec1 %ia% age_group + vacc_status
* age_group,
  data = df_surv_30 %>%
    filter(
      date_censor >= as.Date("2021-12-01"),
      date_atrisk <= as.Date("2021-12-01")
    ))

anova(fit_omicron_30)

plot(anova(fit_omicron_30))
```

© 2022 Eythorsson E et al. *JAMA Network Open.*

```
knitr::kable(summary(fit_omicron_30))

plot(summary(fit_omicron_30))

Predict(fit_omicron_30, time_to_dec1, vacc_status, age_group, fun = plogis) %
>%
  ggplot() +
  geom_vline(aes(xintercept = 30), lty = 2) +
  facet_wrap(~ age_group, nrow = 3) +
  scale_x_reverse(breaks = c(0, 100, 200, 300, 400, 500, 600), limits = c(NA,
0)) +
  scale_y_continuous(labels = scales::percent) +
  labs(
    x = "Days from initial infection to December 1, 2021",
    y = "Proportion reinfected"
  ) +
  theme_bw() +
  theme(legend.position = "none")
```

Based on 60-day reinfection definition (main analysis)

Next, we create a new analysis set where a reinfection is defined as 60 days or longer from the last positive PCR test.

```
df_covid_60 <- df_pcr %>%
  filter(result == "positive") %>%
  group_by(kt) %>%
  arrange(kt, date) %>%
  mutate(
    test_nr = row_number(),
    t = as.numeric(difftime(date, lag(date), units = "days")),
    t = if_else(is.na(t), 0, t),
    cumsum = cumsum(t)) %>%
  ungroup() %>%
  filter(test_nr == 1 | t >= 60) %>%
  mutate(reinfected = if_else(test_nr > 1, 1, 0)) %>%
  left_join(
    df_pcr %>%
      select(kt, date_death) %>%
      distinct()
  )

df_covid_60 <- df_covid_60 %>%
  mutate(age_group = factor(case_when(
    age < 18 ~ "17yr or younger",
    age >= 18 & age <= 29 ~ "18-29yr",
    age >= 30 & age <= 49 ~ "30-49yr",
    age >= 50 & age <= 74 ~ "50-74yr",
    age >= 75 ~ "75yr or older"
  ), levels = c("17yr or younger", "18-29yr", "30-49yr", "50-74yr", "75yr or
```

© 2022 Eythorsson E et al. *JAMA Network Open.*

```
older")
  ))
```

We must ensure that each person is only represented in one line, such that the date column for those who are reinfected represents the date of their first positive PCR test, but the line otherwise refers to their second infection.

```
kt_reinfected_60 <- df_covid_60 %>%
  filter(reinfected == 1) %>%
  pull(kt)

df_surv_60 <- df_covid_60

df_surv_60 <- df_surv_60 %>%
  mutate(
    remove = case_when(
      kt %in% kt_reinfected_60 & reinfected == 0 ~ "remove",
      TRUE ~ "dont_remove"
    )
  ) %>%
  filter(remove != "remove")

df_surv_60 <- df_surv_60 %>%
  mutate(date_censor = case_when(
    reinfected == 1 ~ date,
    date_death <= as.Date("2022-02-13") ~ date_death,
    TRUE ~ as.Date("2022-02-13")
  )) %>%
  left_join(
    df_covid_60 %>%
      filter(kt %in% kt_reinfected_60, reinfected == 0) %>%
      select(kt, date_fix = date)
  ) %>%
  mutate(date = if_else(reinfected == 1, date_fix, date)) %>%
  mutate(
    date_atrisk = date + 60,
    surv_time = as.numeric(difftime(date_censor, as.Date("2021-12-01"), units
= "days"))
  ) %>%
  filter(date_atrisk <= date_censor)
```

Here we add the vaccination data. Because so few individuals had received a booster shot by December 1, 2021 had become reinfected, we dichotomized vaccine status to ≤1 dose and ≥2 doses.

```
df_surv_60 <- df_surv_60 %>%
  mutate(
    time_to_dec1 = as.numeric(difftime(as.Date("2021-12-01"), date, units = "
days"))) %>%
  left_join(
```

© 2022 Eythorsson E et al. *JAMA Network Open.*

```
    df_vacc %>%
      select(-starts_with("age")) %>%
      mutate(across(.cols = starts_with("date"), .fns = ~as.Date(.x)))
  ) %>%
  mutate(vacc_status = factor(case_when(
    !is.na(date_4) & date_4 <= as.Date("2021-12-01") ~ "Two or more vaccine d
oses",
    !is.na(date_3) & date_3 + 14 <= as.Date("2021-12-01") ~ "Two or more vacc
ine doses",
    !is.na(date_3) & date_3 <= as.Date("2021-12-01") & date_3 + 14 >= as.Date
("2021-12-01") ~ "Two or more vaccine doses",
    !is.na(date_2) & date_2 + 14 <= as.Date("2021-12-01") ~ "Two or more vacc
ine doses",
    !is.na(date_2) & date_2 <= as.Date("2021-12-01") & date_2 + 14 >= as.Date
("2021-12-01") ~ "One dose or unvaccinated",
    !is.na(date_1) & date_1 + 14 <= as.Date("2021-12-01") ~ "One dose or unva
ccinated",
    !is.na(date_1) & date_1 <= as.Date("2021-12-01") & date_1 + 14 >= as.Date
("2021-12-01") ~ "One dose or unvaccinated",
    TRUE ~ "One dose or unvaccinated"
  ), levels = c("One dose or unvaccinated", "Two or more vaccine doses")
  )) %>%
  select(-date_4, -date_3, -date_2, -date_1, -name_4, -name_3, -name_2, -name
_1)
```

Number of patients

Number of patients who had their first positive PCR test at least 60 days before 2021-12-01.

```
df_surv_60 %>%
  filter(
    date_atrisk <= as.Date("2021-12-01"),
    date_censor >= as.Date("2021-12-01")
  ) %>%
  count(kt) %>%
  nrow()
```

Number of reinfected patients

Number of patients who had been reinfected using the 60 day definition.

```
df_surv_60 %>%
  filter(
    date_atrisk <= as.Date("2021-12-01"),
    date_censor >= as.Date("2021-12-01")
  ) %>%
  count(reinfected)
```

© 2022 Eythorsson E et al. *JAMA Network Open.*

Sex by reinfection status

```
df_surv_60 %>%
  filter(
    date_atrisk <= as.Date("2021-12-01"),
    date_censor >= as.Date("2021-12-01")
  ) %>%
  count(reinfected, sex)
```

Distribution of age

```
df_surv_60 %>%
  filter(
    date_atrisk <= as.Date("2021-12-01"),
    date_censor >= as.Date("2021-12-01")
  ) %>%
  pull(age) %>%
  quantile()

df_surv_60 %>%
  filter(
    date_atrisk <= as.Date("2021-12-01"),
    date_censor >= as.Date("2021-12-01"),
    reinfected == 0
  ) %>%
  pull(age) %>%
  quantile()

df_surv_60 %>%
  filter(
    date_atrisk <= as.Date("2021-12-01"),
    date_censor >= as.Date("2021-12-01"),
    reinfected == 1
  ) %>%
  pull(age) %>%
  quantile()
```

Time from initial infection

```
df_surv_60 %>%
  filter(
    date_atrisk <= as.Date("2021-12-01"),
    date_censor >= as.Date("2021-12-01")
  ) %>%
  pull(time_to_dec1) %>%
  quantile()
```

Distribution of follow-up times

```
df_surv_60 %>%
  filter(
    date_atrisk <= as.Date("2021-12-01"),
    date_censor >= as.Date("2021-12-01")
  ) %>%
```

© 2022 Eythorsson E et al. *JAMA Network Open.*

```
  pull(surv_time) %>%
  quantile()

df_surv_60 %>%
  filter(
    date_atrisk <= as.Date("2021-12-01"),
    date_censor >= as.Date("2021-12-01"),
    reinfected == 1
  ) %>%
  pull(surv_time) %>%
  quantile()
```

Vaccine status by reinfection

```
df_surv_60 %>%
  filter(
    date_atrisk <= as.Date("2021-12-01"),
    date_censor >= as.Date("2021-12-01")
  ) %>%
  count(reinfected, vacc_status)
```

Vaccine status by age group

```
df_surv_60 %>%
  filter(
    date_atrisk <= as.Date("2021-12-01"),
    date_censor >= as.Date("2021-12-01")
  ) %>%
  count(age_group, vacc_status)
```

Table 1 (main analysis)

```
df_surv_60 %>%
  filter(
    date_atrisk <= as.Date("2021-12-01"),
    date_censor >= as.Date("2021-12-01")
  ) %>%
  count(age_group, reinfected) %>%
  pivot_wider(
    id_cols = age_group,
    names_from = reinfected,
    values_from = n
  ) %>%
  mutate(total = `1`+`0`, prop = `1`/(`1`+`0`)*100) %>%
  select(age_group, total, num_reinfected = `1`, prop) %>%
  kable()
```

Cox regression model

```
covid_datadist <- datadist(
df_surv_60 %>%
  filter(
    date_censor >= as.Date("2021-12-01"),
    date_atrisk <= as.Date("2021-12-01")
```

© 2022 Eythorsson E et al. *JAMA Network Open.*

```
  ) %>%
  select(-date_death))

covid_datadist$limits["Adjust to", "age_group"] <- "18-29yr"
covid_datadist$limits["High:effect", "time_to_dec1"] <- 547
covid_datadist$limits["Low:effect", "time_to_dec1"] <- 91

options(datadist = "covid_datadist")

coxfit_omicron_60 <- cph(Surv(surv_time, reinfected) ~ rcs(time_to_dec1, 5) +
vacc_status + age_group +
                           time_to_dec1 %ia% vacc_status + time_to_dec1 %ia%
age_group + vacc_status * age_group,
                         x = TRUE,
                         y = TRUE,
                         data = df_surv_60 %>%
                           filter(
                             date_censor >= as.Date("2021-12-01"),
                             date_atrisk <= as.Date("2021-12-01")
                           ))

anova(coxfit_omicron_60)

plot(anova(coxfit_omicron_60))

knitr::kable(summary(coxfit_omicron_60))

plot(summary(coxfit_omicron_60))

coxfit_omicron_60 <- cph(Surv(surv_time, reinfected) ~ rcs(time_to_dec1, 5) +
vacc_status + strat(age_group) +
                           time_to_dec1 %ia% vacc_status + time_to_dec1 %ia%
strat(age_group) + vacc_status * strat(age_group),
                         x = TRUE,
                         y = TRUE,
                         data = df_surv_60 %>%
                           filter(
                             date_censor >= as.Date("2021-12-01"),
                             date_atrisk <= as.Date("2021-12-01")
                           ))

survplot_60 <- ggsurvplot(
  fit = survfit(coxfit_omicron_60),
  data = df_surv_60 %>%
    filter(
      date_censor >= as.Date("2021-12-01"),
      date_atrisk <= as.Date("2021-12-01")
    ),
  fun = "event",
  break.x.by = 15,
```

© 2022 Eythorsson E et al. *JAMA Network Open.*

```
  xlim = c(0, 80),
  xlab = "Days from start of Omicron wave (December 1, 2021)",
  ylim = c(0, 0.55),
  ylab = "Cumulative proportion reinfected",
  legend.labs = c(
    "17yr or younger",
    "18-29yr",
    "30-49yr",
    "50-74yr",
    "75yr or older"
  ),
  palette = c(
    "#374E55FF",
    "#DF8F44FF",
    "#00A1D5FF",
    "#B24745FF",
    "#79AF97FF"
  ),
  legend.title = "",
  conf.int = TRUE,
  risk.table = TRUE,
  cumevents = TRUE,
  ggtheme = theme_bw()
)

survplot_60$plot <- survplot_60$plot +
  scale_y_continuous(labels = scales::percent)

survplot_60
```

Logistic regression models

```
fit_omicron_60 <- lrm(
  reinfected ~ rcs(time_to_dec1, 5) + vacc_status + age_group +
    time_to_dec1 %ia% vacc_status + time_to_dec1 %ia% age_group + vacc_status
* age_group,
  data = df_surv_60 %>%
    filter(
      date_censor >= as.Date("2021-12-01"),
      date_atrisk <= as.Date("2021-12-01")
    ))

anova(fit_omicron_60)

plot(anova(fit_omicron_60))

knitr::kable(summary(fit_omicron_60))

plot(summary(fit_omicron_60))

Predict(fit_omicron_60, time_to_dec1, vacc_status, age_group, fun = plogis) %
>%
```

© 2022 Eythorsson E et al. *JAMA Network Open.*

```
  ggplot() +
  geom_vline(aes(xintercept = 60), lty = 2) +
  facet_wrap(~ age_group, nrow = 3) +
  scale_x_reverse(breaks = c(0, 100, 200, 300, 400, 500, 600), limits = c(NA,
0)) +
  scale_y_continuous(labels = scales::percent) +
  labs(
    x = "Days from initial infection to December 1, 2021",
    y = "Proportion reinfected"
  ) +
  theme_bw() +
  theme(legend.position = "none")
```

Based on 90-day reinfection definition (sensitivity analysis)

Finally, we create an analysis set where reinfection is defined to have occur if 90 days or longer had passed since the last positive PCR test.

```
df_covid_90 <- df_pcr %>%
  filter(result == "positive") %>%
  group_by(kt) %>%
  arrange(kt, date) %>%
  mutate(
    test_nr = row_number(),
    t = as.numeric(difftime(date, lag(date), units = "days")),
    t = if_else(is.na(t), 0, t),
    cumsum = cumsum(t)) %>%
  ungroup() %>%
  filter(test_nr == 1 | t >= 90) %>%
  mutate(reinfected = if_else(test_nr > 1, 1, 0)) %>%
  left_join(
    df_pcr %>%
      select(kt, date_death) %>%
      distinct()
  )

df_covid_90 <- df_covid_90 %>%
  mutate(age_group = factor(case_when(
    age < 18 ~ "17yr or younger",
    age >= 18 & age <= 29 ~ "18-29yr",
    age >= 30 & age <= 49 ~ "30-49yr",
    age >= 50 & age <= 74 ~ "50-74yr",
    age >= 75 ~ "75yr or older"
  ), levels = c("17yr or younger", "18-29yr", "30-49yr", "50-74yr", "75yr or
older")
  ))
```

First we must ensure that each person is only represented in one line, such that the date column for those who are reinfected represents the date of their first positive PCR test, but the line otherwise refers to their second infection.

© 2022 Eythorsson E et al. *JAMA Network Open.*

```
kt_reinfected_90 <- df_covid_90 %>%
  filter(reinfected == 1) %>%
  pull(kt)

df_surv_90 <- df_covid_90

df_surv_90 <- df_surv_90 %>%
  mutate(
    remove = case_when(
      kt %in% kt_reinfected_90 & reinfected == 0 ~ "remove",
      TRUE ~ "dont_remove"
    )
  ) %>%
  filter(remove != "remove")

df_surv_90 <- df_surv_90 %>%
  mutate(date_censor = case_when(
    reinfected == 1 ~ date,
    date_death <= as.Date("2022-02-13") ~ date_death,
    TRUE ~ as.Date("2022-02-13")
  )) %>%
  left_join(
    df_covid_90 %>%
      filter(kt %in% kt_reinfected_90, reinfected == 0) %>%
      select(kt, date_fix = date)
  ) %>%
  mutate(date = if_else(reinfected == 1, date_fix, date)) %>%
  mutate(
    date_atrisk = date + 90,
    surv_time = as.numeric(difftime(date_censor, as.Date("2021-12-01"), units
= "days"))
  ) %>%
  filter(date_atrisk <= date_censor)
```

Here we add the vaccination data. Because so few individuals had received a booster shot by December 1, 2021 had become reinfected, we dichotomized vaccine status to ≤1 dose and ≥2 doses.

```
df_surv_90 <- df_surv_90 %>%
  mutate(
    time_to_dec1 = as.numeric(difftime(as.Date("2021-12-01"), date, units = "
days"))) %>%
  left_join(
    df_vacc %>%
      select(-starts_with("age")) %>%
      mutate(across(.cols = starts_with("date"), .fns = ~as.Date(.x)))
  ) %>%
  mutate(vacc_status = factor(case_when(
    !is.na(date_4) & date_4 <= as.Date("2021-12-01") ~ "Two or more vaccine d
oses",
```

© 2022 Eythorsson E et al. *JAMA Network Open.*

```
    !is.na(date_3) & date_3 + 14 <= as.Date("2021-12-01") ~ "Two or more vacc
ine doses",
    !is.na(date_3) & date_3 <= as.Date("2021-12-01") & date_3 + 14 >= as.Date
("2021-12-01") ~ "Two or more vaccine doses",
    !is.na(date_2) & date_2 + 14 <= as.Date("2021-12-01") ~ "Two or more vacc
ine doses",
    !is.na(date_2) & date_2 <= as.Date("2021-12-01") & date_2 + 14 >= as.Date
("2021-12-01") ~ "One dose or unvaccinated",
    !is.na(date_1) & date_1 + 14 <= as.Date("2021-12-01") ~ "One dose or unva
ccinated",
    !is.na(date_1) & date_1 <= as.Date("2021-12-01") & date_1 + 14 >= as.Date
("2021-12-01") ~ "One dose or unvaccinated",
    TRUE ~ "One dose or unvaccinated"
  ), levels = c("One dose or unvaccinated", "Two or more vaccine doses")
  )) %>%
  select(-date_4, -date_3, -date_2, -date_1, -name_4, -name_3, -name_2, -name
_1)
```

Number of patients

Number of patients who had their first positive PCR test at least 90 days before 2021-12-01.

```
df_surv_90 %>%
  filter(
    date_atrisk <= as.Date("2021-12-01"),
    date_censor >= as.Date("2021-12-01")
  ) %>%
  count(kt) %>%
  nrow()
```

Number of reinfected patients

```
df_surv_90 %>%
  filter(
    date_atrisk <= as.Date("2021-12-01"),
    date_censor >= as.Date("2021-12-01")
  ) %>%
  count(reinfected)
```

Sex by reinfection status

```
df_surv_90 %>%
  filter(
    date_atrisk <= as.Date("2021-12-01"),
    date_censor >= as.Date("2021-12-01")
  ) %>%
  count(reinfected, sex)
```

Distribution of age

```
df_surv_90 %>%
  filter(
    date_atrisk <= as.Date("2021-12-01"),
```

© 2022 Eythorsson E et al. *JAMA Network Open.*

```
    date_censor >= as.Date("2021-12-01"),
    reinfected == 0
  ) %>%
  pull(age) %>%
  quantile()

df_surv_90 %>%
  filter(
    date_atrisk <= as.Date("2021-12-01"),
    date_censor >= as.Date("2021-12-01"),
    reinfected == 1
  ) %>%
  pull(age) %>%
  quantile()
```

Time from initial infection

```
df_surv_90 %>%
  filter(
    date_atrisk <= as.Date("2021-12-01"),
    date_censor >= as.Date("2021-12-01")
  ) %>%
  pull(time_to_dec1) %>%
  quantile()
```

Distribution of follow-up times

```
df_surv_90 %>%
  filter(
    date_atrisk <= as.Date("2021-12-01"),
    date_censor >= as.Date("2021-12-01")
  ) %>%
  pull(surv_time) %>%
  quantile()

df_surv_90 %>%
  filter(
    date_atrisk <= as.Date("2021-12-01"),
    date_censor >= as.Date("2021-12-01"),
    reinfected == 1
  ) %>%
  pull(surv_time) %>%
  quantile()
```

Vaccine status by reinfection

```
df_surv_90 %>%
  filter(
    date_atrisk <= as.Date("2021-12-01"),
    date_censor >= as.Date("2021-12-01")
  ) %>%
  count(reinfected, vacc_status)
```

© 2022 Eythorsson E et al. *JAMA Network Open.*

Vaccine status by age group

```
df_surv_90 %>%
  filter(
    date_atrisk <= as.Date("2021-12-01"),
    date_censor >= as.Date("2021-12-01")
  ) %>%
  count(age_group, vacc_status)
```

Table 1 (sensitivity analysis)

```
df_surv_90 %>%
  filter(
    date_atrisk <= as.Date("2021-12-01"),
    date_censor >= as.Date("2021-12-01")
  ) %>%
  count(age_group, reinfected) %>%
  pivot_wider(
    id_cols = age_group,
    names_from = reinfected,
    values_from = n
  ) %>%
  mutate(total = `1`+`0`, prop = `1`/(`1`+`0`)*100) %>%
  select(age_group, total, num_reinfected = `1`, prop) %>%
  kable()
```

Cox regression model

```
covid_datadist <- datadist(
  df_surv_90 %>%
    filter(
      date_censor >= as.Date("2021-12-01"),
      date_atrisk <= as.Date("2021-12-01")
    ) %>%
    select(-date_death))
covid_datadist$limits["Adjust to", "age_group"] <- "18-29yr"
covid_datadist$limits["High:effect", "time_to_dec1"] <- 547
covid_datadist$limits["Low:effect", "time_to_dec1"] <- 91

options(datadist = "covid_datadist")

coxfit_omicron_90 <- cph(Surv(surv_time, reinfected) ~ rcs(time_to_dec1, 5) +
vacc_status + age_group +
                           time_to_dec1 %ia% vacc_status + time_to_dec1 %ia%
age_group + vacc_status * age_group,
                         x = TRUE,
                         y = TRUE,
                         data = df_surv_90 %>%
                           filter(
                             date_censor >= as.Date("2021-12-01"),
                             date_atrisk <= as.Date("2021-12-01")
                           ))
```

© 2022 Eythorsson E et al. *JAMA Network Open.*

```
anova(coxfit_omicron_90)

plot(anova(coxfit_omicron_90))

knitr::kable(summary(coxfit_omicron_90))

plot(summary(coxfit_omicron_90))

coxfit_omicron_90 <- cph(Surv(surv_time, reinfected) ~ rcs(time_to_dec1, 5) +
vacc_status + strat(age_group) +
                           time_to_dec1 %ia% vacc_status + time_to_dec1 %ia%
strat(age_group) + vacc_status * strat(age_group),
                         x = TRUE,
                         y = TRUE,
                         data = df_surv_90 %>%
                           filter(
                             date_censor >= as.Date("2021-12-01"),
                             date_atrisk <= as.Date("2021-12-01")
                           ))

survplot_90 <- ggsurvplot(
  fit = survfit(coxfit_omicron_90),
  data = df_surv_90 %>%
    filter(
      date_censor >= as.Date("2021-12-01"),
      date_atrisk <= as.Date("2021-12-01")
    ),
  fun = "event",
  break.x.by = 15,
  xlim = c(0, 80),
  xlab = "Days from start of Omicron wave (December 1, 2021)",
  ylim = c(0, 0.55),
  ylab = "Cumulative proportion reinfected",
  legend.labs = c(
    "17yr or younger",
    "18-29yr",
    "30-49yr",
    "50-74yr",
    "75yr or older"
  ),
  palette = c(
    "#374E55FF",
    "#DF8F44FF",
    "#00A1D5FF",
    "#B24745FF",
    "#79AF97FF"
  ),
  legend.title = "",
  conf.int = TRUE,
  risk.table = TRUE,
```

© 2022 Eythorsson E et al. *JAMA Network Open.*

```
  cumevents = TRUE,
  ggtheme = theme_bw()
)

survplot_90$plot <- survplot_90$plot +
  scale_y_continuous(labels = scales::percent)

survplot_90
```

Logistic regression models

```
fit_omicron_90 <- lrm(
  reinfected ~ rcs(time_to_dec1, 5) + vacc_status + age_group +
    time_to_dec1 %ia% vacc_status + time_to_dec1 %ia% age_group + vacc_status
* age_group,
  data = df_surv_90 %>%
    filter(
      date_censor >= as.Date("2021-12-01"),
      date_atrisk <= as.Date("2021-12-01")
    ))

anova(fit_omicron_90)

plot(anova(fit_omicron_90))

knitr::kable(summary(fit_omicron_90))

plot(summary(fit_omicron_90))

Predict(fit_omicron_90, time_to_dec1, vacc_status, age_group, fun = plogis) %
>%
  ggplot() +
  geom_vline(aes(xintercept = 90), lty = 2) +
  facet_wrap(~ age_group, nrow = 3) +
  scale_x_reverse(breaks = c(0, 100, 200, 300, 400, 500, 600), limits = c(NA,
0)) +
  scale_y_continuous(labels = scales::percent) +
  labs(
    x = "Days from initial infection to December 1, 2021",
    y = "Proportion reinfected"
  ) +
  theme_bw() +
  theme(legend.position = "none")
```

© 2022 Eythorsson E et al. *JAMA Network Open.*
